# Chitosan Oligosaccharides Alleviate Colitis by Regulating Intestinal Microbiota and PPARγ/SIRT1-Mediated NF-κB Pathway

**DOI:** 10.3390/md20020096

**Published:** 2022-01-24

**Authors:** Congcong Guo, Yue Zhang, Tao Ling, Chongjie Zhao, Yanru Li, Meng Geng, Sailun Gai, Wei Qi, Xuegang Luo, Liehuan Chen, Tongcun Zhang, Nan Wang

**Affiliations:** 1 Key Laboratory of Industrial Fermentation Microbiology, Ministry of Education and Tianjin, College of Biotechnology, Tianjin University of Science and Technology, Tianjin 300457, China; gcongu5413@163.com (C.G.); geby555@163.com (Y.Z.); lingling970614@163.com (T.L.); ZCJ15364948239@163.com (C.Z.); yanrulee930620@mail.tust.edu.cn (Y.L.); gengmeng@mail.tust.edu.cn (M.G.); sailun0728@163.com (S.G.); qiweismiling@126.com (W.Q.); luoxuegang@hotmail.com (X.L.); tony@tust.edu.cn (T.Z.); 2 Tianjin Engineering Research Center of Microbial Metabolism and Fermentation Process Control, Tianjin 300457, China; 3 College of Animal Sciences and Technology, Zhongkai Agricultural Engineering College, Guangzhou 510225, China; chitins@126.com; 4 Guangzhou Youlan Marine Biological Technology Co., Ltd., Guangzhou 510530, China

**Keywords:** chitosan oligosaccharides, ulcerative colitis, PPARγ, SIRT1, NF-κB, intestinal microbiota

## Abstract

Chitosan oligosaccharides (COS) have been shown to have potential protective effects against colitis, but the mechanism underlying this effect has not been fully elucidated. In this study, COS were found to significantly attenuate dextran sodium sulfate-induced colitis in mice by decreasing disease activity index scores, downregulating pro-inflammatory cytokines, and upregulating Mucin-2 levels. COS also significantly inhibited the levels of nitric oxide (NO) and IL-6 in lipopolysaccharide-stimulated RAW 264.7 cells. Importantly, COS inhibited the activation of the NF-κB signaling pathway via activating PPARγ and SIRT1, thus reducing the production of NO and IL-6. The antagonist of PPARγ could abolish the anti-inflammatory effects of COS in LPS-treated cells. COS also activated SIRT1 to reduce the acetylation of p65 protein at lysine 310, which was reversed by silencing SIRT1 by siRNA. Moreover, COS treatment increased the diversity of intestinal microbiota and partly restored the *Firmicutes*/*Bacteroidetes* ratio. COS administration could optimize intestinal microbiota composition by increasing the abundance of *norank_f_Muribaculaceae*, *Lactobacillus* and *Alistipes,* while decreasing the abundance of *Turicibacte*. Furthermore, COS could also increase the levels of propionate and butyrate. Overall, COS can improve colitis by regulating intestinal microbiota and the PPARγ/SIRT1-mediated NF-κB pathway.

## 1. Introduction

Inflammatory bowel disease (IBD), mainly manifested as ulcerative colitis (UC) and Crohn’s disease (CD), is an immune-mediated chronic recurrent gastrointestinal inflammatory disease, and its burden is on the rise globally, including the associated medical and social costs. Currently, the drugs for IBD treatment include aminosalicylic acid, glucocorticoids, antibiotics, immunomodulators, etc., but their therapeutic effect is still unsatisfactory, with large side effects and a high recurrence rate [1], suggesting the necessity to develop new drugs or functional foods beneficial to IBD patients with fewer adverse effects.

Chitosan oligosaccharides (COS), a mixture of oligomers with β-1,4-linked D-glucosamine residues, are derived from the decomposition or deacetylation and degradation of chitin [2]. In recent years, researchers have paid increasing attention to the anti-inflammatory effect of COS, which was shown to downregulate the production of nitric oxide (NO), interleukin-6 (IL-6) and tumor necrosis factor-α (TNF-α) in mouse macrophages stimulated by lipopolysaccharide (LPS) and/or c-interferon [2]. Furthermore, oral COS administration was reported to ameliorate intestinal inflammation in an experimental IBD model [3].

Nuclear factor κB (NF-κB), as the master regulator of inflammation, is activated by inflammatory inducers and translocated into the nucleus to trigger the transcription of downstream target genes, including some inflammatory mediators and cytokines. COS have been reported to repress LPS-induced NF-κB signaling and pro-inflammatory cytokine production in human colonic epithelial cells [2] and intestinal porcine epithelial cells [3]. However, how COS affect the NF-κB pathway remains elusive.

Peroxisome proliferator-activated receptor gamma (PPARγ), a member of the nuclear hormone receptor superfamily, is highly expressed in intestinal epithelial cells and adipocytes, but its expression was lower in the colonic epithelium of UC patients relative to healthy people [4]. Meanwhile, PPARγ is also expressed in macrophages and plays an important role in regulating intestinal inflammation [5]. PPARγ expression loss was observed in lamina propria macrophages of DSS-induced colitis mice [6]. Studies have shown that PPARγ interferes with the function of NF-κB in inflammatory response by directly binding to p65, causing the ubiquitination and degradation of p65, or increasing the expression of Sirtuin 1 (SIRT1) [7]. SIRT1, a NAD^+^-dependent histone deacetylase, has been reported to inhibit the activity of NF-κB via deacetylation of p65 Lys310 [8]. However, whether COS can alleviate colitis by activating the PPARγ/SIRT1-mediated NF-κB signaling pathway is still unclear.

Compared with healthy people, UC patients showed a significant imbalance in intestinal microbiota, including a decrease in bacterial abundance and diversity, an increase in fungal diversity, and a decrease in methanogen diversity [9]. The imbalance of intestinal microecology may destroy the host immune response and intestinal barrier function. COS could inhibit the growth of various bacteria, including *E. coli*, *Salmonella enteritidis*, and *Listeria monocytogenes* [10]. COS treatment in vitro showed a significant increase in the abundance of *Lactobacillus* and *Bifidobacterium* and the concentration of short chain fatty acids (SCFAs) in the cecum of mice [11]. However, the regulatory effect of COS on intestinal microbiota in UC is still not entirely clear.

The purpose of the present study was to explore the protective mechanism of COS against colitis through a mouse model of DSS-induced colitis and a cell model of LPS-induced inflammation. Oral COS administration was found to protect the mice from DSS-induced colitis by suppressing the production of inflammatory factors, preventing the inflammation response via activating PPARγ and SIRT1, inhibiting the acetylation and phosphorylation of NF-κB p65, and optimizing the intestinal microbiota composition.

## 2. Results

### 2.1. Oral COS Administration Alleviated DSS-Induced Colitis in Mice

**Figure 1:**
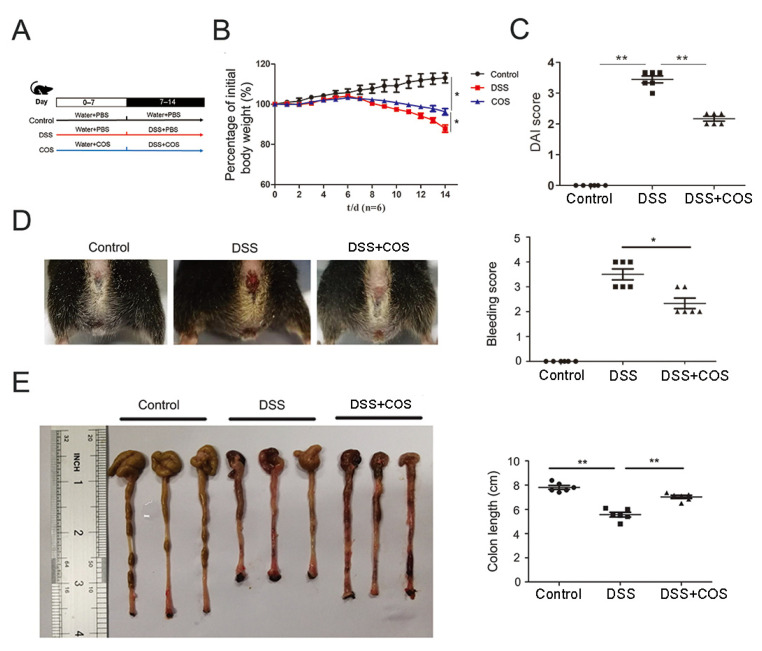
Oral COS administration alleviated the severity of DSS-induced colitis in mice. (**A**) The detailed administration method. Effect of COS treatment on the body weight (**B**), DAI score (**C**), rectal bleeding (**D**) and colon length (**E**) of DSS-induced colitis mice. *, *p* < 0.05, **, *p* < 0.01, *n* = 6.

The therapeutic effect of COS was assessed using a DSS-induced mouse colitis model, with the detailed administration information shown in Figure 1A. Compared with the control group, the DSS-treated group showed significant (*p* < 0.05) body weight loss at the end of the two weeks, and this body weight loss was partially (*p* < 0.05) recovered in the COS-treated group (Figure 1B). Meanwhile, significantly reduced DAI scores were also obviously (*p* < 0.01) rescued by oral COS administration (Figure 1C). In Figure 1D,E, it was shown that DSS-treated mice exhibited significant rectal bleeding and shortened colon length (*p* < 0.01), whereas oral COS administration obviously reduced the bleeding and colon shortening (*p* < 0.05, *p* < 0.01).

**Figure 2:**
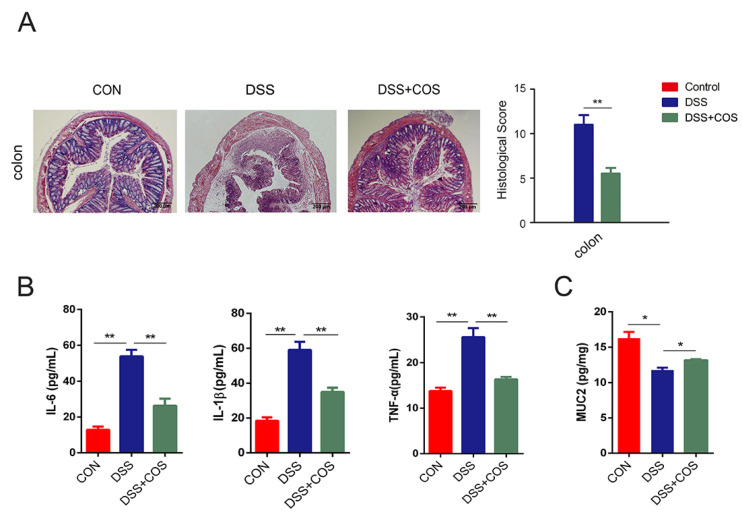
Oral COS administration ameliorated intestinal mucosal damage and reduced the inflammatory response in DSS-induced colitis mice. (**A**) Images of H&E-stained colon and ileum sections from DSS-treated mice with or without COS administration. The scale bar is 200 μm or 100 μm, as indicated in the respective images. Pathological scores of these tissues were calculated. (**B**) The levels of IL-6, IL-1β and TNF-α in serum were detected by ELISA. (**C**) The level of MUC2 in colonic tissue was detected by ELISA. *, *p* < 0.05, **, *p* < 0.01, *n* = 6.

H&E staining showed severe damage of crypts, loss of goblet cells, infiltration of mononuclear cells, and even formation of serious ulcers in the colons and ileums of DSS-induced colitis mice (Figure 2A). However, oral COS administration significantly reduced inflammation and ameliorated structural damage. The effects of COS on the levels of pro-inflammatory cytokines in the serum were further investigated by ELISA. In Figure 2B, the levels of IL-6, IL-1β and TNF-α were shown to significantly (*p* < 0.01) increase in the DSS-treated group versus the control group, while their levels were seen to be obviously (*p* < 0.01) inhibited by COS treatment. Furthermore, COS treatment upregulated the levels of mucin-2 (MUC 2) (*p* < 0.05), which is a major intestinal O-glycosylated protein secreted by goblet cells, suggesting the improvement of intestinal barrier function (Figure 2C).

### 2.2. COS Inhibited Inflammation in LPS-Stimulated RAW 264.7 Cells and DSS-Induced Colitis Mice by Activating PPARγ/SIRT1 and Inhibiting the NF-κB Pathway

**Figure 3:**
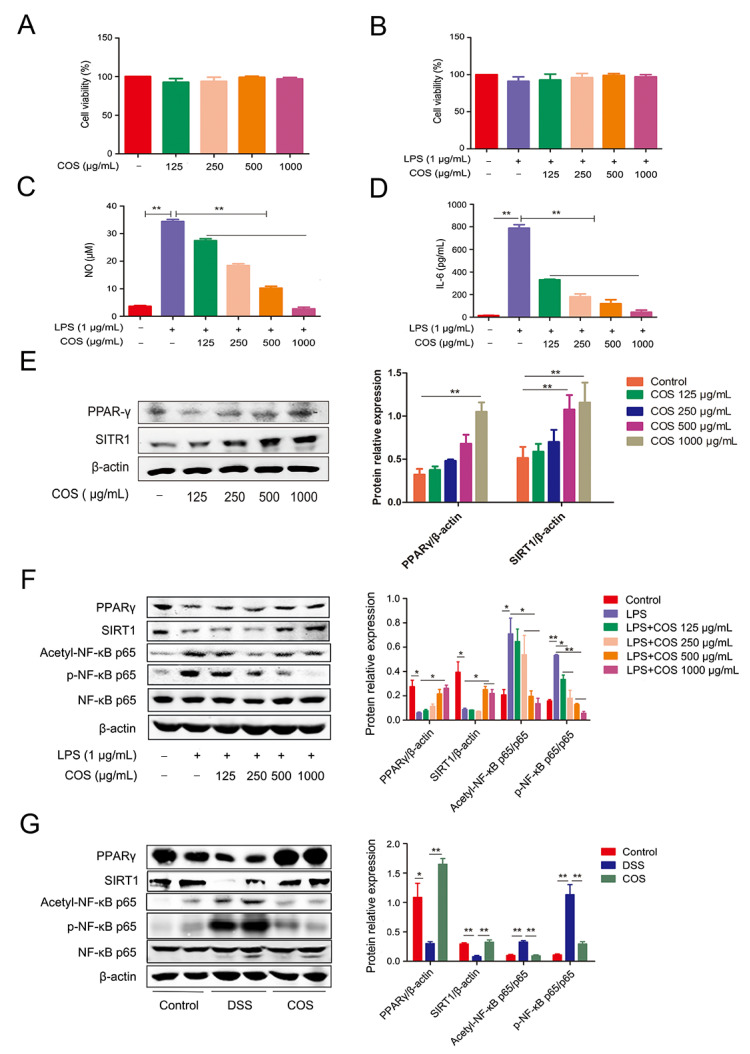
COS inhibited the LPS-induced production of NO and IL-6 in RAW 264.7 cells and inhibited DSS-induced colitis in mice by activating PPARγ/SIRT1 and inhibiting the NF-κB pathway. RAW 264.7 cells were pre-treated with COS for 2 h and cultured in the presence of LPS for another 24 h. Cell viability in the absence (**A**) or presence (**B**) of LPS was determined by MTT assay. The levels of NO (**C**) and IL-6 (**D**) in the culture medium were determined by NO assay kit or ELISA, respectively. (**E**) RAW264.7 cells were treated with COS for 24 h, and the expressions of PPARγ and SIRT1 were measured by western blotting. (**F**) RAW264.7 cells were pretreated with COS for 24 h and stimulated with LPS for 1 h. The proteins were extracted and subjected to western blot analysis. (**G**) Western blotting analysis of the expression of relative genes in the colonic tissues of COS-treated mice with colitis. *, *p* < 0.05, **, *p* < 0.01, *n* = 3.

The anti-inflammatory effect of COS was further investigated using RAW 264.7 cells in vitro. The cellular viability was not significantly affected by COS at 125, 250, 500 and 1000 µg/mL in the absence/presence of LPS (Figure 3A,B). However, COS pretreatment was found to attenuate the LPS-induced increase of NO and IL-6 in a concentration-dependent manner (*p* < 0.01) (Figure 3C,D).

To explore the potential molecular mechanism of COS in alleviating colitis, we tested the effect of COS on PPARγ/SIRT1 and NF-κB signaling pathway in LPS-stimulated RAW 264.7 cells and in the colonic tissues of DSS-induced colitis mice. COS alone treatment could upregulate the protein level of PPARγ at 1000 μg/mL (*p* < 0.01) and also increased the protein level of SIRT1 at 500 and 1000 μg/mL (*p* < 0.01) (Figure 3E). In Figure 3F, LPS-stimulated RAW 264.7 cells showed a significantly (*p* < 0.05) low expression of PPARγ relative to the control, while COS pretreatment could upregulate the protein level of PPARγ, especially at 1000 μg/mL (*p* < 0.05). Similarly, LPS stimulation significantly (*p* < 0.05) reduced the protein level of SIRT1, whereas COS treatment at 500 and 1000 μg/mL increased (*p* < 0.05) the SIRT1 expression in LPS-stimulated cells. Moreover, COS treatment significantly (*p* < 0.05; *p* < 0.01) inhibited the LPS-induced phosphorylation of NF-κB p65 and repressed the acetylation of p65 at Lys310 (*p* < 0.05).

In Figure 3G, consistent with the results of the in vitro experiment, DSS treatment significantly (*p* < 0.05; *p* < 0.01) downregulated the levels of PPARγ and SIRT1, which was significantly ameliorated by COS treatment. Furthermore, COS treatment blocked the activation of the NF-κB p65 signaling pathway, as indicated by the lower phosphorylation and acetylation of p65 (*p* < 0.05, *p* < 0.01). These results showed that COS can alleviate DSS-induced colitis in mice by activating PPARγ/SIRT1 and inhibiting NF-κB signaling pathway.

### 2.3. COS Inhibited the Activation of NF-κB Signaling Pathway via Activating PPARγ, Thus Reducing the Production of NO and IL-6

**Figure 4:**
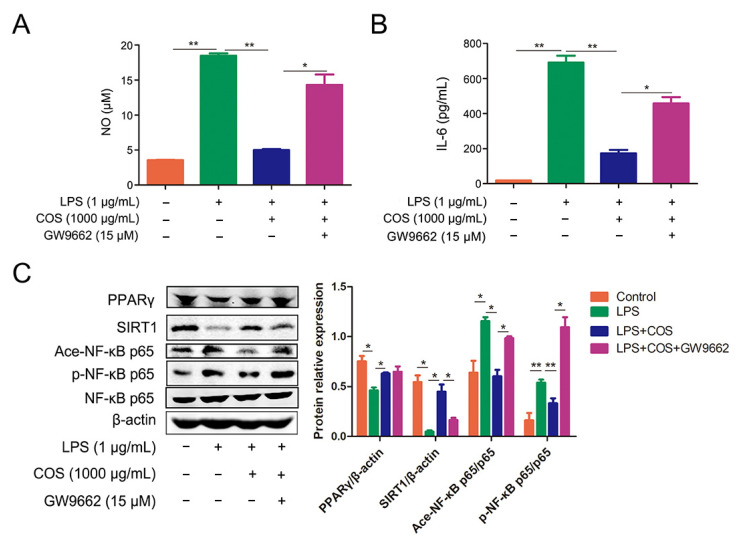
COS inhibited the NF-κB signaling pathway and reduced the production of NO and IL-6 by activating PPARγ. RAW 264.7 cells were pre-incubated with PPARγ antagonist GW9662 (15 µM) for 1 h, followed by treatment with COS for 2 h, and incubation with LPS for another 24 h. The levels of NO (**A**) and IL-6 (**B**) in the culture medium were determined by NO assay kit and ELISA. (**C**) RAW264.7 cells were incubated with GW9662 (15 µM) for 1 h, followed by treatment with COS (1000 µg/mL) for another 24 h, and stimulated with LPS for 1 h. Protein levels were determined by the western blotting assay. *, *p* < 0.05, **, *p* < 0.01, *n* = 3.

To further confirm whether the anti-inflammatory effect of COS is PPARγ-dependent, GW9662, a specific PPARγ antagonist, was introduced in this study. COS treatment was shown to significantly reduce the production of NO and IL-6 in LPS-stimulated RAW 267.4 cells, which was almost reversed by adding GW9662 (*p* < 0.05, *p* < 0.01) (Figure 4A,B). Meanwhile, COS administration was also found to upregulate the expression of PPARγ and SIRT1 in LPS-stimulated cells, whereas the presence of GW9662 blocked the expression of SIRT1, suggesting that inhibition of PPARγ could reduce COS-induced activation of SIRT1 (*p* < 0.05) (Figure 4C). Furthermore, as a response to LPS, GW9662 significantly weakened COS-mediated inhibition of acetylation and phosphorylation of NF-κB p65 in cells (*p* < 0.05, *p* < 0.01) (Figure 4C). These results demonstrated that PPARγ can mediate the COS-induced inhibition of the NF-κB signaling pathway.

### 2.4. COS-Mediated Inhibition of NF-κB Signaling Pathway Was Dependent on SIRT1

**Figure 5:**
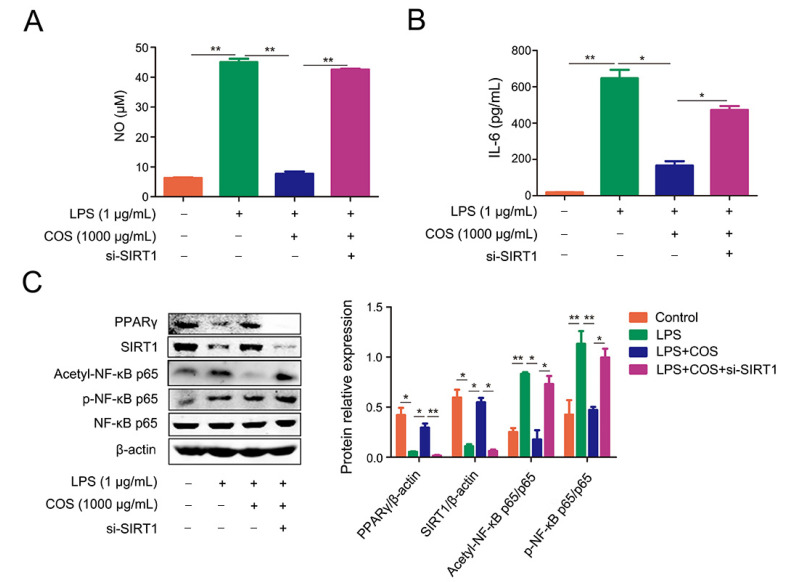
COS-mediated inhibition of the NF-κB signaling pathway was dependent on SIRT1. RAW 264.7 cells were transfected with si-SIRT1 for 5 h, followed by treatment with COS for 2 h, and incubation with LPS for another 24 h. The levels of NO (**A**) and IL-6 (**B**) in the culture medium were determined by NO assay kit and ELISA, respectively. (**C**) RAW264.7 cells were transfected with si-SIRT1 or si-Control for 6 h, incubated with COS for 24 h, and then stimulated with LPS for 1 h. Protein levels were determined by the western blotting assay. *, *p* < 0.05, **, *p* < 0.01, *n* = 3.

To study whether the anti-inflammatory effect of COS is SIRT1-dependent, the siRNA of SIRT1 was transfected into cells to knock down the expression of SIRT1. As shown in Figure 5A,B, the LPS-induced production of NO and IL-6 was attenuated by COS, which was significantly reversed by knockdown of SIRT1 (*p* < 0.05, *p* < 0.01). si-SIRT1 transfection weakened the upregulation of PPARγ expression induced by COS, suggesting that SIRT1 could also affect the expression of PPARγ (*p* < 0.01) (Figure 5C). Accordingly, COS treatment repressed the acetylation and phosphorylation of NF-κB p65 in LPS-stimulated cells, which was blocked by si-SIRT1 (*p* < 0.05, *p* < 0.01) (Figure 5C). The data indicated that COS produced an anti-inflammatory effect through SIRT1-mediated deacetylation of NF-κB p65.

### 2.5. Oral COS Administration Optimized Intestinal Microbiota Composition in Mice with Colitis

**Figure 6:**
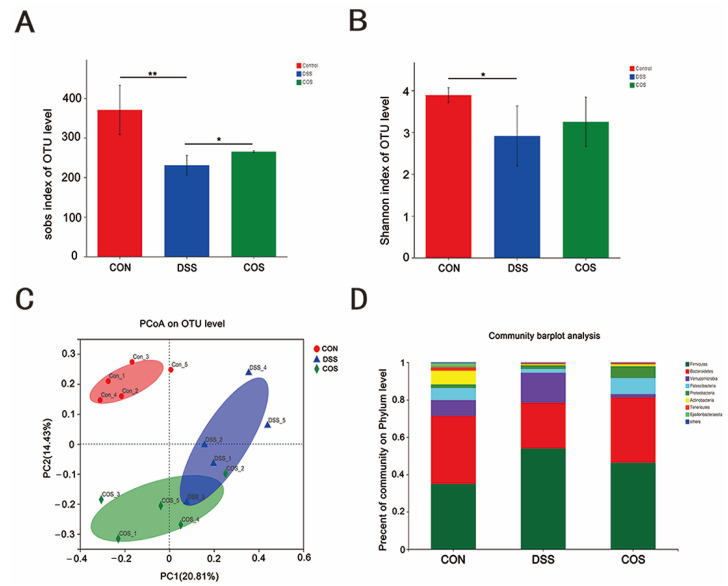
COS administration upregulated the abundance and diversity of gut microbiota and modulated the overall structure of the gut microbiota in mice with colitis. The α-diversity of microbial communities in cecal contents was assessed in terms of the Sobs index (**A**) and Shannon index (**B**). (**C**) Principal co-ordinate analysis (PCoA) of gut microbial communities in cecal contents. (**D**) Bar chart of the bacterial community composition at the phylum level. *, *p* <0.05, **, *p* < 0.01, *n* = 5.

To investigate the effect of COS on DSS-induced gut microbiota dysbiosis, we analyzed the microbiota composition of colonic contents isolated from healthy and DSS-administered mice. Compared with healthy mice, DSS-induced colitis mice showed a significant reduction in the α-diversity of microbial communities (*p* < 0.05, *p* < 0.01), which was largely reversed after COS oral administration (Figure 6A,B). PCoA revealed three clearly separate clusters for the Control group, DSS group and COS group (Figure 6C). As shown in Figure 6D, compared with the Control group, DSS treatment caused a significant expansion of *Firmicutes* and a reduction of *Bacteroidetes,* suggesting an increased *Firmicutes*/*Bacteroidetes* (*F*/*B*) ratio, and this ratio was ameliorated after oral COS administration.

**Figure 7:**
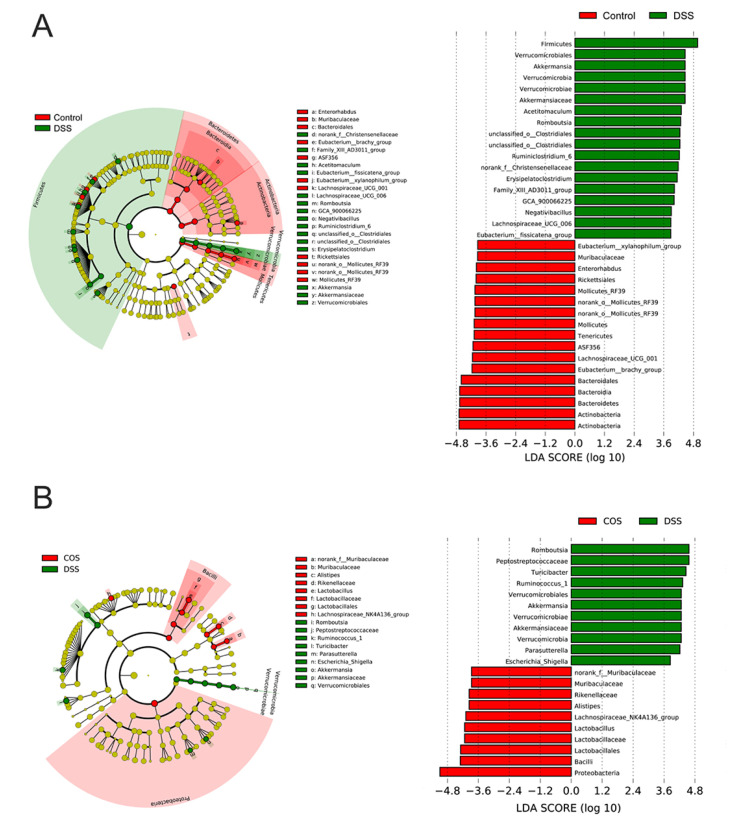
Cladogram showing the polygenetic distribution of the bacterial lineages associated with different groups. (**A**) LEfSe analysis between control and DSS groups. (**B**) LEfSe analysis between DSS and COS groups. LEfSe provided the features that are differential bacterial taxa ranking according to the effect size. Different color nodes represent the microbial groups that are significantly enriched in corresponding groups and have a significant influence on the differences between groups (Control group (red) and DSS group (green) in Figure 7A; COS group (red) and DSS group (green) in Figure 7B). The light-yellow node indicates the microbial groups that have no significant difference in different groups or have no significant influence on the differences between groups. Only the taxa with an LDA score higher than 3.5 were shown.

**Figure 8:**
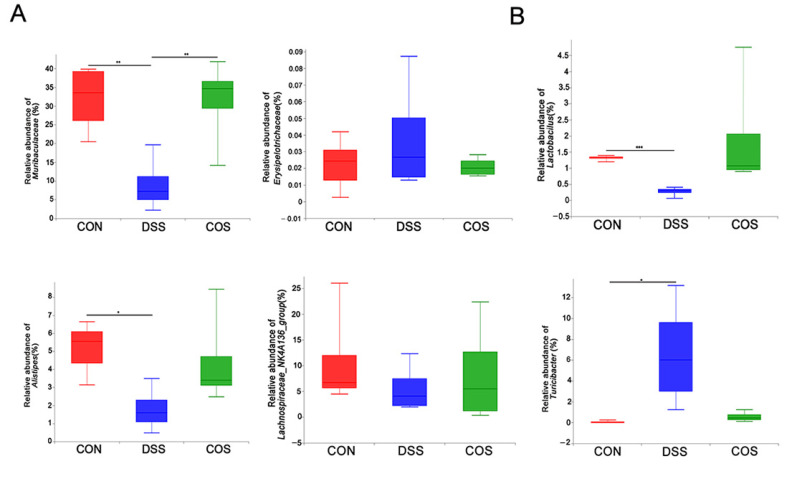
COS administration restored the abundance of certain bacteria in mice with colitis. (**A**) Relative abundances of specific microbial populations at the family level. (**B**) Relative abundances of specific microbial populations at the genus level. * *p* < 0.05, ** *p* < 0.01, *** *p* < 0.001, *n* = 5.

Besides Circos analysis, we also performed the linear discriminant analysis effect size (LEfSE) analysis from the phylum to genus level. The DSS group showed significant enrichment in *Turicibacter*, *Romboutsia*, *Prevotellaceae*, *Verrucomicrobiales, Ruminiclostridium* and *Escherichia_Shigella*, in contrast to the dominance of *norank_f_Muribaculaceae* and *Lactobacillus* in the COS group (Figure 7A,B). Compared with the Control group, DSS treatment group showed a decreased abundance of *Muribaculaceae* (*p* < 0.01) and an increased abundance of *Erysipelatoclostridium* at the family level, whereas COS treatment reversed this change and significantly restored the abundance of *Muribaculaceae* (*p* < 0.01) (Figure 8A). COS treatment also partly decreased the abundance of *Erysipelatoclostridium*, but not significantly. At the genus level, DSS treatment decreased the abundance of *Lactobacillus* (*p* < 0.001), *Alistipes* (*p* < 0.05) and *Lachnospiraceae_NK4A136_group* and increased the abundance of *Turicibacter* (*p* < 0.05) (Figure 8B). Variations in these bacterial genera were partly reversed by oral COS administration.

### 2.6. Oral COS Administration Enhanced the Production of SCFAs in Mice with Colitis

**Figure 9:**
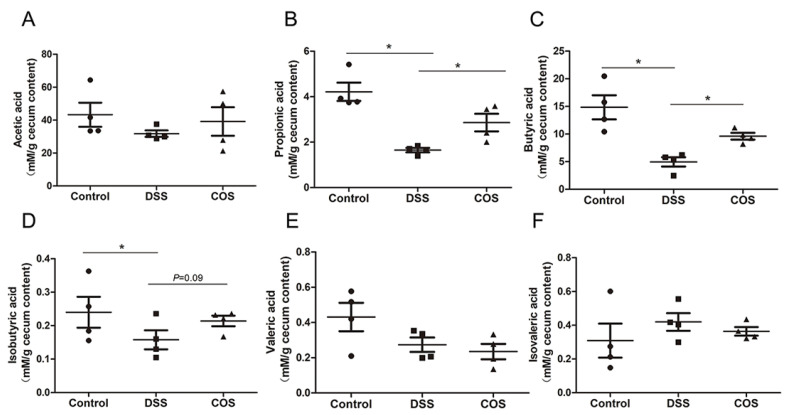
COS upregulated the concentrations of short chain fatty acids in the contents of the cecum. The levels of acetic acid (**A**), propionic acid (**B**), butyric acid (**C**), isobutyric acid (**D**), valeric acid (**E**) and isovaleric acid (**F**) in cecal contents. *, *p* < 0.05, *n* = 4.

SCFAs are the major end products of bacterial fermentation and play an important role in resistance to inflammation and protection of intestinal mucosal integrity. DSS treatment significantly reduced the contents of propionic acid, butyric acid and isobutyric acid, whereas oral COS administration remarkably (*p* < 0.05) upregulated the concentration of propionic acid and butyric acid in cecal contents (Figure 9B–D), with no significant variation in the concentration of acetic acid, valeric acid and isovaleric acid (Figure 9A,E,F).

### 2.7. Correlation of COS-Modified Intestinal Microbiota with Intestinal Injury, Intestinal Barrier, Inflammatory Cytokine Levels, and SCFA Levels

**Figure 10:**
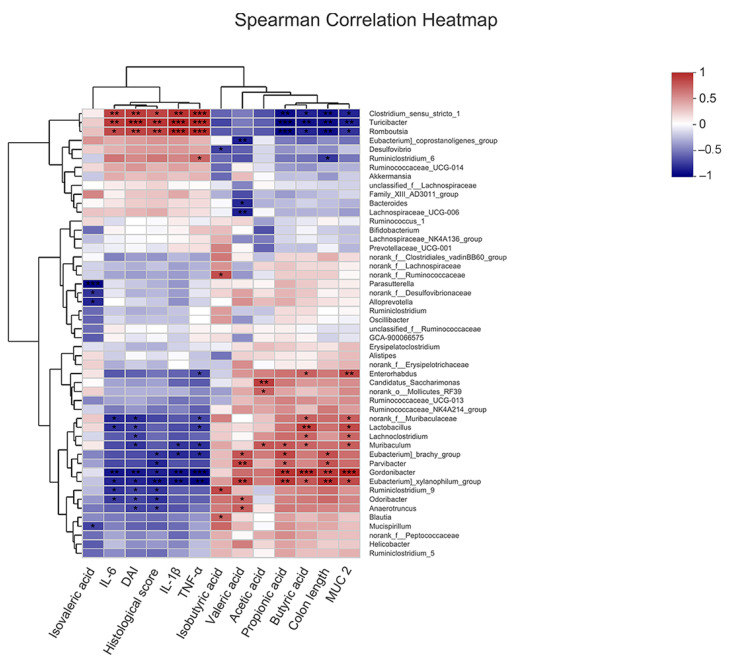
Spearman analysis of the correlation of microbiota at the genus level with intestinal injury, intestinal barrier, inflammatory cytokine levels and SCFA levels. The top 50 most abundant genera in each sample were used for the hierarchical clustering and heatmap analyses based on the Spearman correlation coefficient. The red and blue blocks represent the positive and negative correlations, respectively, and the color grade shows the correlation degree. *, *p* < 0.05, **, *p* < 0.01, ***, *p* < 0.001, *n* = 4.

To further explore the protective effects of COS-modified gut microbiota against UC, the relationship of the top 50 most abundant genera in all samples with colon length, DAI score, histological score, inflammatory cytokine levels, MUC2 levels and SCFA levels were analyzed by Spearman correlation coefficient. As shown in Figure 10, *norank_f_Muribaculaceae* and *Lactobacillus*, which were enriched in the COS group, displayed a positive correlation with the levels of MUC2 and butyric acid, but a negative correlation with DAI score and the levels of IL-6 and TNF-α (*p* < 0.05, *p* < 0.01). *Turicibacter* and *Romboutsia*, which were enriched in the DSS group, were positively correlated with the DAI score, histological score and the levels of the inflammatory factors, but negatively correlated with the levels of MUC2, propionic acid, butyric acid and colon length (*p* < 0.05, *p* < 0.01, *p* < 0.001). These results indicated that the alterations in intestinal microbiota induced by COS could play essential roles in alleviating intestinal inflammation by reducing intestinal injury, inhibiting inflammatory responses, improving intestinal barrier and upregulating SCFA levels.

## 3. Discussion

Although COS has been reported to alleviate DSS-induced colitis, the molecular mechanisms underlying its anti-inflammatory benefits are poorly understood. In this study, we investigated how COS affect the NF-κB signaling pathway and intestinal microbiota in LPS-stimulated RAW 264.7 cells and DSS-induced colitis mice.

COS are not digested by gastrointestinal enzymes after ingestion but are readily absorbed through the intestinal epithelium into the blood and have systemic biological effects in organisms [12,13,14,15]. In an in vitro study, water-soluble chitosans could be transported through a monolayer of Caco-2 cells, and the absorption rate was negatively correlated with the MW of chitosans [12]. In vivo, the plasma concentration of chitosans reached its peak value 30 min after oral administration. Low-MW chitosans (MW = 3.8 kDa) have the greatest plasma concentration after oral administration vs. high-MW chitosans (MW = 230 kDa), which have almost no uptake. COS are the degraded products of chitosans. COS, which have greater solubility and low viscosity with relatively smaller molecular sizes, are more easily absorbable in in vivo systems [13]. COS can be absorbed from the small intestine into the blood and distributed to the liver, kidney and spleen [14]. The absorbed COS can be degraded by lysozymes in the blood, liver, kidney and urine, whereas the unabsorbed COS can reach the distal intestine and be utilized by gut microbiota.

The DSS-induced colitis model is widely used due to its rapidity, simplicity, reproducibility and controllability, as well as its many similarities with human UC [16]. DSS is a water-soluble sulfated polysaccharide with a molecular weight range of 5–1400 kDa. Generally, DSS with a molecular weight of 36–50 kDa is employed to induce colitis. Murine colitis induced by administration of 40–50 kDa DSS in drinking water most closely resembles human UC [17]. However, the mechanism by which DSS initiates colitis remains unclear. DSS is thought to be toxic to the colonic epithelial cells due to its highly negative charge, and disrupts the integrity of the mucosal barrier, resulting in increased colonic epithelial permeability [16]. Furthermore, accumulated evidence confirmed that the possible pathogenic mechanism induced by DSS might not be involved in the acquired immune system [18,19]. Using natural killer cell-deficient and T- and B-cell deficient mice, they found that DSS administration equally caused colonic inflammation, suggesting that these cells might not be critical for DSS-induced colitis, and this model may be suitable for studying the contribution of the innate immune system in the generation of colitis. Thus, the murine macrophage cell line RAW 264.7 cells was used to study the anti-inflammatory effect of COS in vitro.

Consistent with previously reported results [2], our results also demonstrated that COS could inhibit the inflammatory response in LPS-stimulated RAW 264.7 macrophages and alleviate ulcerative colitis in mice. COS have been reported to attenuate inflammation and related inflammatory injury by inhibiting NF-κB-mediated inflammation and apoptosis in LPS-stimulated intestinal epithelial cells (IECs) in vitro and in the colonic tissues of mice with colitis in vivo [20,21]. Here, our results showed that COS prevented LPS-stimulated production of IL-6 and NO in macrophages in vitro by inhibiting the NF-κB signaling pathway. Similar to the above, COS also repressed the NF-κB signaling pathway in the colonic tissues of mice with ulcerative colitis.

Although PPARγ has been known as a nuclear receptor expressed in adipose tissue to be involved in the regulation of insulin resistance, it is also highly expressed in the colon tissues, including IECs, macrophages and lymphocytes, and is involved in the regulation of inflammation and mucosal damage in UC lesions [22]. The reduced expression of PPARγ was observed in the colonic epithelium of UC patients [23]. PPARγ-deficient mice exhibited a significantly greater degree of injury after intestinal ischemia-reperfusion (I/R), while the activation of PPARγ blocked I/R-induced intestinal injury [24]. PPARγ also plays a protective role against intestinal tissue injury induced by DSS [25] or 2,4,6-trinitrobenzene sulfonic acid (TNBS) [5]. Activation of PPARγ significantly reduces the levels of IL-1β, IL-6 and TNF-α in LPS-stimulated macrophages by repressing the NF-κB signaling pathway [26], suggesting that the protective effects of PPARγ is associated with inhibition of NF-κB activity. In this study, COS upregulated the expression of PPARγ to inhibit the activation of the NF-κB pathway in murine macrophages, leading to the decreased production of IL-6 and NO. Meanwhile, the antagonist of PPARγ could abolish the anti-inflammatory effects of COS in LPS-treated cells, suggesting that the regulation of COS on the NF-κB signaling pathway could be at least partly mediated by PPARγ. Consistent with the results in vitro, oral COS administration ameliorated the severity of UC symptoms, coupled with the increased expression of the PPARγ protein. In Caco-2 cells, the expression of PPARγ could be induced by the constitutively active form of Toll-like receptor 4 (TLR4) [27]. COS has been shown to upregulate the expression of TLR4 in IPEC-J2 cells [3], suggesting COS could activate PPARγ through TLR4.

COS can act as immunostimulants to elicit immune cell responses when acting alone, but it can function as an anti-inflammatory agent in the presence of LPS [2,28,29,30,31,32,33]. COS alone can enhance NO production, phagocytosis of macrophages, and the generation of pro-inflammatory cytokines, such as IL-1β, IL-6 and TNF-α, through the NF-κB, AP-1, MAPK and PI3K/Akt signaling pathways [29,30,31]. However, pretreatment with COS could attenuate the LPS-induced macrophage inflammatory response. One of the mechanisms is that COS can compete with LPS to bind TLR4 and inhibit the binding of LPS to TLR4, to diminish the corresponding inflammation signal transduction, including the NF-κB, MAPK, and other pathways [32,33]. Shi et al. also confirmed that TLR4 could act as a receptor for COS, because COS alone upregulated the expression of TLR4 [2].

Numerous data suggest that the activation of TLRs modulates the expression of PPARγ [27,34,35]. However, the results may be contradictory, and the mechanisms of their control are poorly understood. Activation of inflammatory responses by LPS or other pro-inflammatory particles causes a decrease in the expression of PPARγ [34,35,36]. LPS activated TLR4 and reduced PPARγ expression and function in peritoneal macrophages and macrophage cell lines, including RAW264.7 cells [35]. LPS had no effect on PPARγ expression in macrophages from TLR4 knockout mice, whereas LPS inhibited PPARγ expression in cells that restored TLR4 expression [35]. Upon activation of TLR4, NF-κB inhibited PPARγ mRNA synthesis and protein expression in RAW264.7 cells [35]. However, other studies have also confirmed that the expression of PPARγ could be induced by the constitutively active form of TLR4 [27]. Therefore, COS may activate the expression of PPARγ through other signaling pathways mediated by TLR4. Consistent with our results, COS have been reported to upregulate the expression of PPARγ in HepG2 cells [37] and 3T3-L1 cells [38] in vitro and high-fat diet (HFD)-fed C57BL/6J mice in vivo [37]. Thus, COS could increase the expression of PPARγ through TLR4.

Bendixen et al. reported that IL-4 suppressed the receptor activator of NF-κB ligand (RANKL)-induced osteoclast formation from RAW264.7 cells, which could be blocked by GW9662 [39]. IL-4 suppressed RANKL-induced activation of NF-κB, but the inhibitory effect of IL-4 on NF-κB could not be reversed with GW9662. They considered that GW9662 had no effect on NF-κB because IL-4 did not cause the upregulation of PPARγ1 expression in RAW264.7 cells. Another possible explanation was that there might be additional PPAR-independent complexity in the action of IL-4. In our study, COS could upregulate the expression of PPARγ and inhibit the phosphorylation of NF-κB, which could be reversed by GW9662. This result was coincident with most findings that PPARγ could inhibit the activation of the NF-κB signal and the release of inflammatory factors, which could be blocked by GW9662 [40,41,42,43]. Bergenin, acting as an agonist of PPARγ, inhibited LPS-mediated macrophage activation in RAW264.7 cells [40]. Compared with the control group, both the LPS group (only LPS) and the GW9662 group (LPS + GW9662) induced NF-κB activation and exhibited similar results in RAW264.7 cells [40]. The addition of bergenin inhibited LPS-mediated NF-κB activation and this inhibitory effect could be reversed by GW9662. Similar to the above, in our study, COS inhibited LPS-induced NF-κB activation, which could be prevented by GW9662.

Besides direct interaction with p65 and thus inactivation of p65 NF-κB, PPARγ also promotes the inactivation of NF-κB by indirect effects. Recent studies have suggested that activation of PPARγ could upregulate the expression of SIRT1, a class III histone deacetylase (HDAC3), in RAW 264.7 cells [7]. Schug et al. reported that NF-κB activity could be activated by deletion of SIRT1 in macrophages [44]. SIRT1 could deacetylate p65 protein at lysine 310 and inhibit the transactivation capacity of p65, thereby suppressing the expression of pro-inflammatory genes [45]. In this study, we showed for the first time that COS activated SIRT1 to reduce the acetylation of p65 protein at lysine 310. Given that the PPARγ antagonist could reverse COS-induced upregulation of SIRT1, COS-mediated SIRT1 activation may depend on PPARγ. Silencing SIRT1 by siRNA increased NF-κB p65 acetylation and IL-6 expression in cells exposed to COS and LPS, suggesting that the anti-inflammatory effect of COS was mediated by SIRT1.

Imbalance of intestinal microbiota could cause UC in humans and experimental colitis in mice [46]. The F/B ratio represents the proportion of *Firmicutes* to *Bacteroidetes*. Increased F/B ratio has been suggested as an indicator of several pathological conditions [47]. Recent studies have revealed the relationship between a high abundance of *Firmicutes* and IBD risk [48], and a reduced abundance of *Bacteroidetes* was observed in IBD patients [49]. The gut microbiota is associated with immune function. *Bacteroidetes*, specifically *Bacteroides fragilis*, induces the production of IL-10 by stimulating Th1 and Treg cell responses and protects mice from pathogen-induced colitis [50]. Our result was consistent with the previous finding that DSS treatment resulted in the decreased diversity and richness of intestinal microbiota and the increased ratio of *Firmicutes*/*Bacteroidetes* [51]. Importantly, COS treatment increased the diversity and richness of intestinal microbiota and partly restored the *F*/*B* ratio.

Reduced abundance of *Muribaculaceae* was reported in mice with colitis [51]. *Norank_f_Muribaculaceae* is positively correlated with the expression of barrier function genes in neonatal piglets [52]. Meanwhile, *Alistipes* has been found to protect mice from colitis [53], and reduced enrichment of *Alistipes* was observed in IBD patients and UC mice [54]. *Alistipes* is also shown to be negatively correlated with DAI score, pathological score, and pro-inflammatory cytokine level in UC mice [11]. Consistent with these reports, our results confirmed that DSS treatment decreased the abundance of *Alistipes* and *norank_f_Muribaculaceae*, which can be curbed or reversed by COS administration.

Previous studies showed that *Turicibacter* was increased in mice with DSS-induced colitis [55] or in mice with AOM/DSS-induced colorectal cancer (CRC) [11]. COS protected mice from CRC by reducing the abundance of *Turicibacter*. Similar to these findings, our results confirmed that *Turicibacter* was significantly enriched in the DSS group, and administration of COS corrected this disorder. Furthermore, *Turicibacter* was found to be positively correlated with TNF-α, IL-6, IL-1β, DAI score and histological score and negatively correlated with colon length.

At the genus level, we observed a drastic reduction in *Lactobacillus* (SCFA-producing bacteria) in DSS-induced colitis mice. *Lactobacillus* has been reported to ameliorate colitis by improving intestinal barrier function and modulating gut mucosal immune responses [56,57,58]. In UC mice, the abundance of *Lactobacillus* was negatively correlated with the DAI score [59]. Another SCFA-producing bacteria, *Lachnospiraceae_NK4A136_group*, was negatively correlated with inflammation [60,61]. SCFAs, the beneficial metabolites in the human gut, especially butyrate, are the main energy source of intestinal epithelial cells and play a crucial role in regulating innate immunity, inhibiting inflammatory effects, and maintaining intestinal micro-environmental homeostasis [62,63]. Downregulation of intestinal SCFAs in UC tends to cause an increase in intestinal pH, imbalance of intestinal microbiota and aggravation of inflammatory symptoms. In this study, COS treatment was shown to enhance the abundance of *Lactobacillus* and *Lachnospiraceae_NK4A136_group*, as well as the levels of butyrate and propionate.

Together, COS ameliorated intestinal inflammation by activating PPARγ/SIRT1 and inhibiting the NF-κB signal pathway. The inhibitory effect of COS on the activation of the NF-κB signaling pathway depended on its activation of PPARγ and SIRT1. Furthermore, oral administration of COS improved the diversity and composition of the gut microbiota.

## 4. Materials and Methods

### 4.1. Materials and Reagents

COS (polymerization degree: 2–10; molecular weight: 322–1610 Da; degree of deacetylation: 90%) was provided by Guangzhou Youlan Marine Biotechnology Co., Ltd. (Guangzhou, China). DSS (enteritis model grade, molecular mass, 36,000–50,000 Da) was purchased from MP Biomedicals (Solon, OH, USA). LPS, which was extracted from *Escherichia coli* O55:B5, was purchased from Santa Cruz Biotechnology (Santa Cruz, CA, USA). GW9662 (a PPARγ antagonist) was purchased from MCE (Middlesex County, NJ, USA). The small interfering RNA (siRNA) duplexes for SIRT1 (si-SIRT1) and control siRNA (si-Control) were designed and synthesized by RiboBio Co. (Guangzhou, China). The NO assay kit was purchased from Beyotime (Shanghai, China); Mouse IL-6, IL-1β and TNF-α enzyme-linked immunosorbent assay (ELISA) kits were purchased from R&D Systems (Minneapolis, MN, USA). Mouse Mucin2 (MUC2) ELISA kit was purchased from CUSABIO (Wuhan, China). The anti-NF-κB p65, anti-phospho-NF-κB p65 (Ser536) and anti-PPARγ antibodies were obtained from Santa Cruz Biotechnology (Santa Cruz, CA, USA). Anti-NF-κB p65 (acetyl K310) antibody was obtained from Abcam (Cambridge, UK). Anti-SIRT1 (IF3) antibody was purchased from Cell Signaling Technology Inc. (CST) (Boston, MA, USA). Anti-β-actin antibody was purchased from UtiBody (Tianjin, China). The secondary antibodies used were IRDye-680LT goat anti-rabbit IgG or IRDye-800CW goat anti-mouse IgG (LI-COR Corporate, Lincoln, NE, USA).

### 4.2. Animal Test

Male C57BL/6 mice (8 weeks old) were obtained from the Experimental Animal Center of Military Medical College (Beijing, China) and maintained under standard conditions for seven days of acclimatization before use for experimentation. All animal procedures were performed in accordance with the National Institutes of Health Guide for the Care and Use of Laboratory Animals (NIH Publications No. 8023, revised 1978) and approved by the Animal Ethics Committee of Tianjin University of Science and Technology (Approval No. 2020305CB). These mice were randomly divided into three groups (*n* = 8): control group, DSS group, and COS group. Mice in the DSS group or COS group were orally administered PBS or COS (300 mg/kg/d) for 7 consecutive days, followed by 2.5% (*w/v*) DSS in drinking water for another 7 days. The body weight of each mouse was recorded daily, and the disease activity index (DAI) scores were calculated by monitoring clinical manifestations, such as body weight, stool consistency and rectal bleeding, as previously described [64].

At the end of the experiment (14 days), the mice were fasted for 12 h and sacrificed under anesthesia with sodium isopentobarbital. Blood samples were collected via retroorbital bleeding, followed by centrifugation to separate serum at 1500 r/min for 30 min at 4 °C. Colon and ileum samples were dissected, fixed in 4% buffered formalin and embedded in paraffin to provide sections for hematoxylin-eosin (H&E) staining.

### 4.3. Cell Culture, Cell Treatment and Transfection

RAW 264.7 cells were cultured in Dulbecco’s modified Eagle’s medium (DMEM) containing 10% fetal bovine serum (FBS), penicillin (100 U/mL) and streptomycin (100 μg/mL) in a 5% CO_2_ atmosphere at 37 °C. COS were dissolved at different concentrations in the culture medium. After seeding into 96-well plates or 6-well plates at a density of 2 × 10^5^ cells/mL, the cells were pre-incubated with COS (0, 125, 250, 500, 1000 µg/mL) for 2 h and then cultured in the presence or absence of 1 µg/mL LPS for another 24 h or 48 h, followed by subsequent experiments.

For siRNA transfection, cells were transfected with si-SIRT1 or si-Control for 6 h using Turbofect Transfection Reagent, followed by removing the supernatant and adding fresh medium. After 24 h of culture, the cells were used for subsequent experiments.

### 4.4. Cell Viability Assay

The effects of COS on the viability of RAW 264.7 cells with/without LPS stimulation were evaluated by the MTT assay. Briefly, after treating the cells as described above, 10 µL of MTT solution (5 mg/mL in PBS) was added to each well. After 4 h of incubation, the supernatants were removed, and DMSO was added to each well to dissolve the formazan crystals. The absorbance was measured at 490 nm.

### 4.5. Determination of the Levels of Nitric Oxide, Cytokines, and MUC2

The production of NO in cellular supernatants was detected by an NO assay kit. The levels of IL-6, IL-1β and TNF-α in cellular supernatants and serum samples isolated from animals were detected using an ELISA kit, and the levels of Mucin2 (MUC2) in colonic tissues were detected using an ELISA kit as instructed by the manufacturers.

### 4.6. Western Blot Analysis

The extracted proteins were loaded to SDS-PAGE and transferred to a nitrocellulose membrane. Next, the membrane was blocked with 5% non-fat milk for 1 h at room temperature (RT), followed by incubation with different primary antibodies overnight at 4 °C (anti-p65, anti-phospho-p65 and anti-PPARγ, anti-acetyl-p65, anti-SIRT1 and anti-β-actin) and then with secondary antibodies for two hours at RT (IRDye-680RD IgG or IRDye-800CW IgG). The specific bands were analyzed using an Odyssey Infrared Imaging System (Li-COR Biosciences). Densitometric analysis was performed using Image J software.

### 4.7. 16S rRNA Sequencing and Analysis

Sequencing analysis was performed by the Majorbio Technology Co., Ltd. (Shanghai, China). Total genomic DNA was extracted from the colonic contents. The V3–V4 hypervariable regions of the bacterial 16S rRNA gene were amplified with primers F (5′-ACTCCTACGGGAGGCAGCAG-3′) and R (5′-GGACTACHVGGGTWTCTAAT-3′) in a thermocycler PCR system (GeneAmp 9700, ABI, Waltham, MA, USA). After purification, the amplicons were pooled in equimolar and paired-end sequenced (2 × 300) on an Illumina MiSeq platform (Illumina, San Diego, CA, USA) according to the standard protocols of the Majorbio Bio-Pharm Technology Co. Ltd (Shanghai, China).

### 4.8. Detection of Short Chain Fatty Acid Levels

The levels of SCFAs were detected by gas chromatography 7890A (Agilent, Palo Alto, CA, USA) under the following conditions: column, HP-FFAP (60 mm * 320 μm * 0.50 μm, Agilent, Palo Alto, CA, USA); injection temperature, 250 °C; injection volume, 1 μL; column flow rate, 2 mL/min; split ratio, 1:1. The initial column temperature was set at 90 °C and held for 6 min, then up to 200 °C at 10 °C/min and hold for 10 min. The detector temperature was set as 250 °C, the hydrogen flow rate as 40 mL/min, and the air flow rate as 450 mL/min.

### 4.9. Statistical Analysis

All statistical analyses were performed using either one-way ANOVA or Student’s t-test in GraphPad Prism 6.0 (Graph Pad Software Inc., San Diego, CA, USA). Data were presented as the mean ± SD, and significant statistical differences between groups were determined at *p* < 0.05 and *p* < 0.01.

## 5. Conclusions

Therefore, COS may prevent or treat colitis by suppressing the production of inflammatory factors, preventing the inflammation response via activating PPARγ and SIRT1, inhibiting the acetylation and phosphorylation of NF-κB p65, and optimizing the intestinal microbiota composition. These results facilitate our understanding of the protective mechanism of COS against colitis and suggest the potential of COS as a functional food or drug for the prevention and treatment of colitis.

## Data Availability

The data presented in this study are available on request from the corresponding author.
